# Plant Protease Inhibitors as Emerging Antimicrobial Peptide Agents: A Comprehensive Review

**DOI:** 10.3390/pharmaceutics16050582

**Published:** 2024-04-24

**Authors:** Mónica G. Parisi, Brenda Ozón, Sofía M. Vera González, Javier García-Pardo, Walter David Obregón

**Affiliations:** 1 Instituto de Ecología y Desarrollo Sustentable (INEDES, CONICET-UNLu) and Departamento de Ciencias Básicas, Universidad Nacional de Luján, Ruta 5 y Avenida Constitución, Luján B6700, Buenos Aires, Argentina; mparisi@unlu.edu.ar; 2 Centro de Investigación de Proteínas Vegetales (CIProVe) and Departamento de Ciencias Biológicas, Facultad de Ciencias Exactas, Universidad Nacional de La Plata, 47 y 115 s/N, La Plata B1900, Buenos Aires, Argentina; brendaozon@biol.unlp.edu.ar (B.O.); alu-sveragonzalez@exactas.unlp.edu.ar (S.M.V.G.); 3 Institut de Biotecnologia i de Biomedicina (IBB) and Departament de Bioquímica i Biologia Molecular, Universitat Autònoma de Barcelona, 08193 Bellaterra, Barcelona, Spain

**Keywords:** plant protease inhibitors, antibacterial compounds, antimicrobial activity, antimicrobial peptides, antibiotic resistance, antifungal agents, cysteine-rich peptides

## Abstract

Antimicrobial peptides (AMPs) are important mediator molecules of the innate defense mechanisms in a wide range of living organisms, including bacteria, mammals, and plants. Among them, peptide protease inhibitors (PPIs) from plants play a central role in their defense mechanisms by directly attacking pathogens or by modulating the plant’s defense response. The growing prevalence of microbial resistance to currently available antibiotics has intensified the interest concerning these molecules as novel antimicrobial agents. In this scenario, PPIs isolated from a variety of plants have shown potential in inhibiting the growth of pathogenic bacteria, protozoans, and fungal strains, either by interfering with essential biochemical or physiological processes or by altering the permeability of biological membranes of invading organisms. Moreover, these molecules are active inhibitors of a range of proteases, including aspartic, serine, and cysteine types, with some showing particular efficacy as trypsin and chymotrypsin inhibitors. In this review, we provide a comprehensive analysis of the potential of plant-derived PPIs as novel antimicrobial molecules, highlighting their broad-spectrum antimicrobial efficacy, specificity, and minimal toxicity. These natural compounds exhibit diverse mechanisms of action and often multifunctionality, positioning them as promising molecular scaffolds for developing new therapeutic antibacterial agents.

## 1. Introduction

Natural products (NPs) have long served as a source of active ingredients for a broad array of pharmaceutical applications, owing to their extensive chemical and structural diversity and generally low toxicity [1,2]. Predominantly sourced from plants, these compounds exhibit a wide range of biological mechanisms, offering novel activities and biological properties essential for the development of nutraceuticals, pharmacologically active substances, phytosanitary agents, and other economically valuable products [3,4,5,6].

The increasing emergence of multidrug-resistant (MDR) bacteria is a critical global problem [7,8,9]. From the One Health perspective, this can be considered one of the most significant challenges for the biomedical field, animal health, and phytosanitary services [10,11,12,13]. Indeed, it has recently been estimated that antimicrobial resistance (AMR) was directly responsible for 1.27 million global deaths and contributed to 4.95 million deaths, affecting countries in all regions and at all income levels, being higher in low- and middle-income countries, thus producing significant economic costs (WHO) [14].

In this scenario, the search for natural therapeutic agents, especially as alternatives to synthetic antibiotics, remains a fervent area of research and development within the scientific community. Notably, in the last 35 years, natural compounds have been the basis for over 35% of drugs approved by the Food and Drug Administration (FDA) (https://www.fda.gov/drugs) [15]. This trend is expected to continue, with predictions suggesting that natural and biopharmaceutical products, including small chemical compounds, peptides, and proteins, could form the majority of our pharmacotherapeutic tools in the near future [16].

In the plant kingdom, antimicrobial compounds act as the primary defense mechanism against pathogenic microorganisms. In this context, numerous plant antimicrobial molecules capable of inhibiting a number of human pathogens have been isolated from various plant sources [17,18,19,20]. These natural compounds include primary metabolites essential for plant growth, substance transport, photosynthesis, respiration, nutrient assimilation, flowering, and fruit ripening (i.e., proteins, lipids, and carbohydrates) and secondary plant metabolites (i.e., terpenes, polyphenols, and carotenoids), which do not participate in fundamental physiological processes, although they exhibit diverse bioactive chemical scaffolds. In addition to these small chemical compounds, a variety of proteins and peptides with promising antimicrobial properties have also been isolated and characterized so far. These include chitinases, β-1,3-glucanases, thaumatin-like proteins, endoproteases, peroxidases, ribonuclease-like proteins, γ-thionins, plant defensins, oxalate oxidases, oxalate oxidase-like proteins, proteases, and peptide protease inhibitors (PPIs) [21,22]. During the last two decades, up to eighteen PPIs with antimicrobial activity have been described so far. Among them, several PPIs have demonstrated broad-spectrum efficacy against a wide range of pathogens, including bacteria, fungi, and viruses [23,24,25,26]. These include Kunitz- and Trypsin-type inhibitors from plants belonging to the *Solanaceae*, *Fabaceae*, and *Moringaceae* families.

This work aims to offer a comprehensive review of plant-derived PPIs and their potential as novel antibacterial and antifungal agents, exploring their mechanisms of action, efficacy against various microbial strains, and the potential for their application as innovative antimicrobial peptide agents. Ultimately, we also discuss future directions of this field.

## 2. Antimicrobial Peptides (AMPs)

**Figure 1:**
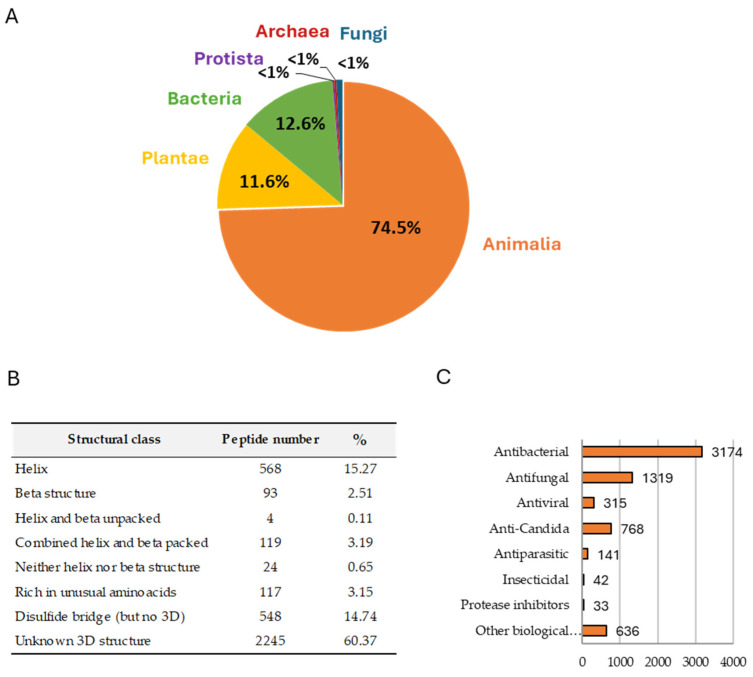
Composition and Diversity of Antimicrobial Peptides (AMPs). (**A**–**C**) These panels summarize the data from the Antimicrobial Peptide Database (APD3) (http://aps.unmc.edu/AP/) [29]. As of January 2024, APD3 has cataloged 3940 AMPs, categorized by (**A**) biological origin, (**B**) secondary structural characteristics, and (**C**) the spectrum of biological activity. In panel (**B**), Unknown 3D structure refers to AMPs for which the three-dimensional structures have not yet been determined.

The extensive research has brought many novel antimicrobial candidates into clinical and pre-clinical development [27,28]. An important number of these molecules are antimicrobial peptides (AMPs), a diverse group of small molecules synthesized and ubiquitously distributed in plants and other living organisms. Due to antimicrobial potential and immunomodulatory capacities, research on AMPs has steadily increased in the last decade. Currently, over 3940 AMPs have been reported so far, including 3146 natural AMPs of different natural sources, including 383 bacteriocins/peptide antibiotics from bacteria; 5 from archaea; 8 from protists; 29 from fungi; 250 from plants; and 2463 from animals, according to the latest update of the Antimicrobial Peptide Database (APD3) (http://aps.unmc.edu/AP/) [29] (Figure 1). While the majority of AMPs have been identified in animals and plants, those isolated from archaea and protists include a set of relevant molecules, such as halocins, that have acquired specific adaptations to extreme environments. These adaptations could translate into robust stability and efficacy under conditions that are challenging for other antimicrobial compounds. The activity and mechanism of these and other AMPs have been thoroughly reviewed elsewhere [30,31,32,33]; therefore, this review will specifically focus on the recent developments in plant-derived AMPs, particularly those with protease inhibition activity.

Plant AMPs are generally classified based on their tridimensional structure, their sequence, and the presence of disulfide bonds, such as thionins, defensins, hevein-like peptides, knottins, stable-like peptides, lipid transfer proteins, snakins, and cyclotides [24,34,35,36,37]. Thionins are essential peptides found in monocots and dicots, with sizes ranging from 45 to 48 amino acid residues and a molecular weight of around 5 kDa [36]. They are classified into two groups: α/β-thionins and γ-thionins, according to amino acid sequence homology, three-dimensional structural similarity, and disulfide bridge positions. Regarding the antimicrobial activity of thionins, antibacterial [38,39], antifungal, anti-larvicidal, and in vitro cytotoxic effects against mammal cell cultures have been reported [24,40].

**Figure 2:**
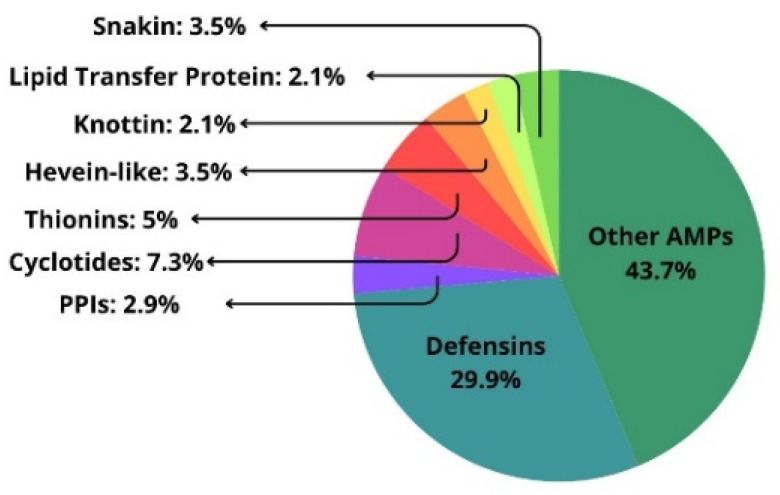
Relative abundance of AMPs available on the Antimicrobial Plant Database (https://aps.unmc.edu/) [29].

Defensins constitute one of the largest groups of AMPs (Figure 2). They have been shown to disrupt cellular functions by binding extracellularly to the cell wall components, membrane targets, and/or to specific membrane lipids such as sphingolipids or phospholipids or binding to cell surface targets and membrane lipids [41]. An important aspect of AMP functionality involves the modulation of the host plant’s defense mechanisms. In this regard, some AMPs have been found to prime the plant’s immune response, preparing it for a more robust defense against subsequent pathogen attacks. This priming effect boosts the plant’s innate immune system, enhancing its responsiveness through the modulation of various signaling cascades [42]. Hevein-like peptides are basic peptides containing 29–45 amino acid residues stabilized by 3–5 disulfide bonds, with a conserved chitin-binding domain (SXFGY/SXYGY), which is associated with antifungal activity [43]. These molecules were first identified in the latex of *Hevea brasiliensis* and are responsible for defense against a wide range of pathogenic fungi based on the interaction of the hevein domain with fungal chitin present in the cell, causing damage to the fungal cell.

Plant knottins are peptides with approximately 30 amino acid residues, the smallest plant AMPs, involving three disulfide bonds (cysteine knot motifs) and two conformations, cyclic and linear, showing high thermal stability and resistance to chemicals and proteolytic action [44]. Knottins are known as “promiscuous peptides” based on their multiple biological functions: they can bind to cell membranes, K^+^ and Na^+^ channels in membranes, and acid-sense channels; are highly cytotoxic to human cells [45]; and have antimicrobial activity against bacteria, fungi, viruses, and insects via interacting with membranes to exert their activities.

The α-hairpinin family represents a class of Lys/Arg-rich plant defense peptides, with special Cys motifs (i.e., XnC1X3C2XnC3X3C4Xn, where X is any amino acid different from cysteine), to form a highly characteristic helix–loop–helix secondary structure with two antiparallel α-helices [46] stabilized by two disulfide bonds in the tertiary structure. This AMP family shows antibacterial, antifungal, trypsin-inactivating, and ribosome-inactivating activities [46,47].

Lipid transfer proteins (LTPs) are small cationic peptides (7 to 10 kDa), composed of a conservative pattern of eight Cys residues and four disulfide bonds that stabilize a tight tertiary fold with hydrophobic cavities. LTPs inhibit the growth of fungi and bacteria, thus participating in plant defense systems [48].

The snakin class contains 12 cysteine residues (cysteine-rich), the largest number of disulfide bonds and are constitutively or inducibly expressed via biotic or abiotic stress in different organs, such as the roots, stem, leaves, flowers, and seeds. Snakins inhibit both fungal (e.g., *Magnaporthe grisea*, *Fusarium solani*, and *Botrytis cinerea*) and bacterial (e.g., *Dickeya dadantii*, *Ralstonia solanacearum*, and *Sinorhizobium meliloti*) growth [49].

Cyclotides are long-chain cyclic peptides (28–37 amino acids) consisting of six loops with six conserved cysteine residues, stabilized via three disulfide bonds. They are anionic AMPs with high resistance to thermal and chemical denaturation as well as proteolytic degradation, making them potential therapeutic agents as antitumor, anti-HIV, insecticidal, and antibacterial agents [50,51].

In this context, it is known that around 3% of AMPs are plant PPIs (Figure 2) with proven effectiveness in inhibiting the growth of a variety of pathogenic bacterial and fungal strains [36]. Not surprisingly, many of these AMPs share common structural and functional properties with plant PPIs, including their ability to alter the integrity of microbial cell membranes, a mechanism crucial for their function [36,52,53,54]. This positions this set of natural peptides as promising molecules for developing novel therapeutics against MDR bacteria.

## 3. Mechanism of Antimicrobial Action of Plant AMPs

Typically, AMPs are characterized by their low molecular weight, displaying less than 100 amino acids with a positive net charge at physiological pH, due to the presence of lysine and arginine residues and a high proportion (≥30%) of hydrophobic residues [32,55]. These characteristics render them highly cationic molecules that can interact with the phospholipid and lipopolysaccharide layers found in many types of bacteria, leading to pore formation, membrane destabilization, and rapid cell death [25,32,56,57,58]. At present, several mechanisms have been reported on cationic AMPs that cause cell membrane damage by interacting with the negatively charged phospholipid layer and lipopolysaccharides of the bacterial membrane, causing pores, membrane destabilization, and rapid cell death [25,56,57,58]. For Gram-negative bacteria, the AMP needs to permeabilize the outer membrane before it can access the cytoplasmic membrane. Conversely, for Gram-positive bacteria, the AMP can directly diffuse through the peptidoglycan layer via nano-sized pores.

**Figure 3:**
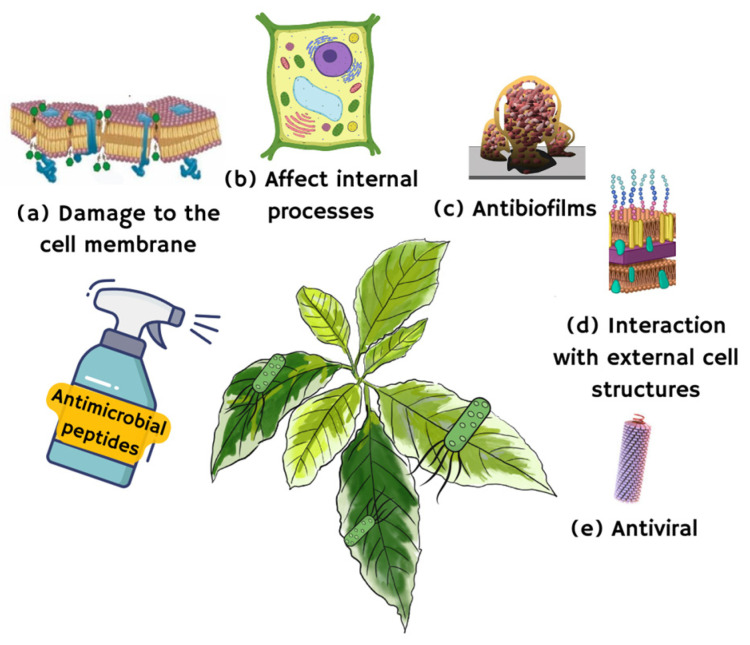
Mechanisms of action of antimicrobial peptides. (**a**) Damage of cell membranes, causing cell lysis. (**b**) Interference with internal cellular processes. (**c**) Inhibition of biofilm formation. (**d**) Interaction with external cell structures such as lipopolysaccharides, fimbriae, or flagella in bacteria or chitin in fungi. (**e**) Inhibition of virus attachment or replication [59].

AMPs can also mediate their action by acting as lipid transfer proteins. Through this mechanism, these proteins directly interact with fungal membrane lipids, effectively blocking pathogen penetration into the host cell and, thus, preventing infection [59]. Furthermore, many AMPs can specifically bind to the sphingolipid and phospholipid bilayers of the cell wall and/or the cell membrane of fungi, being able to enter or remain on the outside of the fungal cell. Regardless of their possible absorption, antifungal peptides can affect intracellular targets, causing, among other actions, the production of ROS, programmed cell death, mitochondrial dysfunction, the alteration of cation homeostasis, ATP efflux, cell cycle impairment, and autophagy, among others [29]. They can penetrate the target cell by breaking the membrane and interfering with protein or nucleic acid synthesis or cell division or by inhibiting protease activity [53,60]. For instance, heveins bind chitin in fungi [43] and some defensins interact with eukaryotic target proteins to target intracellular functions [41]. Another mechanism of action is priming the plant by inducing defense responses against pathogens due to the modulation of different cascades in their immune system (Figure 3) [61].

## 4. Peptide Protease Inhibitors (PPIs) Derived from Plants

Protease inhibitors (PIs) are regulatory molecules of a proteinaceous or non-proteinaceous nature, ubiquitously distributed in animals, plants, and across microbial species. Those of a proteinaceous nature are typically known as PPIs. Based on the type of protease they inhibit, PPIs can be categorized into six main classes: serine protease inhibitors (SPIs), cysteine protease inhibitors (CPIs), aspartyl protease inhibitors (APIs), metalloprotease inhibitors (MPIs), glutamate protease inhibitors (GPIs), and threonine protease inhibitors (TPIs) [62,63,64].

PPIs play a central role in controlling the activity of target proteases, often inhibiting their excessive and uncontrolled activity in both normal and pathological conditions [65]. This regulation is essential for activating coenzymes and releasing biologically active polypeptides [66,67]. In the case of mammals, the presence of PPIs in plasma is closely associated with the regulation of critical proteolytic cascades, including blood coagulation and complement activation [66,68,69,70]. Moreover, proteolysis is involved in many vital biological functions, such as immunity, blood coagulation, cell cycle regulation, and tissue morphogenesis [71,72]. In this context, PPIs have been exploited by the pharmaceutical industry for different applications, including drug discovery and diagnosis [22].

**Figure 4:**
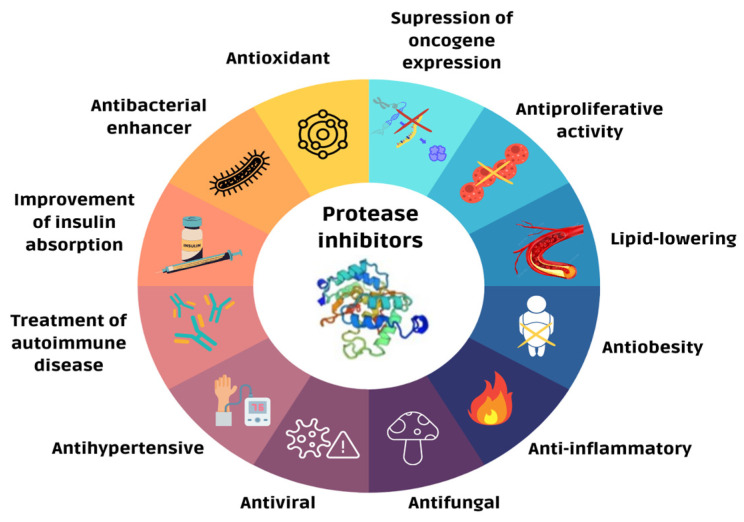
Plant peptide protease inhibitors and their potential applications in biomedicine.

Notably, several low-molecular PPIs from plants contain a high content of cysteine residues that form disulfide bridges that confer resistance to heat treatment, extreme pH or ionic forces, and proteolysis [62,73,74,75,76,77]. Owing to their multifunctionality and remarkable physicochemical properties, PPIs have found a wide array of applications in biotechnology and biomedicine, including their use as antimicrobial agents [21,78,79,80] (Figure 4). Despite their high stability, the presence of multiple cysteine residues involved in disulfide bridges often complicates the production of these molecules, requiring a comprehensive understanding of their folding mechanisms and three-dimensional structure [81,82].

## 5. Plant-Derived Peptide Protease Inhibitors with Antimicrobial Properties

Over the last 15 years, there has been a notable increase in the reports on novel PPIs with antimicrobial properties isolated from various plant sources [22,83,84,85]. An important number of these PPIs have been linked to antimicrobial roles in a physiological context. This is the case of the systemin hormone, an 18-amino acid peptide that plays a critical role in the defense against pathogens and is a key part of the jasmonic acid signaling pathway that triggers PPI expression [73]. In the *Solanaceae* family, the prosystemin mechanism involves the expression of genes predominantly associated with metallo carboxypeptidase inhibitors, cysteine PPIs, and trypsin PPIs, including Kunitz-type PPIs [74,86].

PPIs isolated from a variety of plants have shown potential in inhibiting the growth of pathogenic bacteria, protozoans, and fungal strains (Table 1) [21]. As introduced above, the antimicrobial activity of many plant PPIs is typically mediated through the ability of these molecules to alter the permeability of bacterial cytoplasmic membranes. This toxic action is facilitated via the cationic nature of these peptides, which bind to cell membranes through interactions between positive and negative charges, causing destabilization and the formation of temporary cavities or disruptions. Such damage leads to the leakage of cellular contents and ultimately results in bacterial death [67,75,85]. In addition to their antimicrobial actions, it is also common that these molecules simultaneously target diverse metabolic processes, which has fueled interest in applying these molecules for diverse biomedical applications.

### 5.1. Plant Protease Inhibitors with Antibacterial Activity

Potatoes (*Solanum. tuberosum* spp.) have historically been a rich source of plant PPIs with antimicrobial (antifungal and antibacterial) activities against a wide variety of agricultural and clinical pathogens [100]. A prototypical example is the Kunitz-type serine PPI, Potide-G, isolated from the tubers of *S. tuberosum* L. cv. Golden Valley, which has been shown to be effective in inhibiting the growth of diverse human pathogens, including *S. aureus* and *L. monocytogenes*, through the regulation of extracellular enzymes related to nutrition. This inhibitor showed a strong potency with minimum inhibitory concentration (MIC) values lower than 30 µg/mL, demonstrating a potency comparable to established antibiotics [26]. Similarly, PG-2, a peptide isolated from the potato tubers of cv. Gogu Valley, exhibited antibacterial activity against *S. aureus* and other pathogens, displaying minimal cytotoxic effects against human red blood cells [92].

A significant number of studies have reported on the action mechanisms of trypsin inhibitors on the bacterial membrane. A trypsin PPI called API was purified from *A. amara* seeds, showing antimicrobial activity against *P. aeruginosa* and *B. subtilis,* being useful as a potential antibacterial component [87]. In 2018, Martins et al. [88] discovered a Bowman–Birk PPI (called LzaBBI) from *L. auriculata* seeds that exhibits robust antibacterial activity against *S. aureus*. This 14.3 kDa thermostable protein, similar to other Bowman–Birk inhibitors, demonstrates mixed-type inhibitory activity against trypsin and chymotrypsin. Scanning electron microscopy experiments unequivocally revealed that LzaBBI compromises *S. aureus* membrane integrity, possibly through oxidative stress and reactive oxygen species (ROS) production, leading to cell lysis. In a previous study, Satheesh and Murugan [83] identified a 14.3 kDa trypsin PPI from *C. grandis* leaves that effectively inhibits several pathogenic bacteria, including *S. aureus*. Similarly, a 4 kDa trypsin inhibitor from *C. fistula* leaves, called Fistulin, demonstrated significant antibacterial activity, paralleling the efficacy of streptomycin sulfate by inhibiting microbial proteases [89]. Additionally, a new Kunitz-type inhibitor was isolated from *R. frangula* leaves. This inhibitor displayed a potent inhibition against several proteases from Bacillus sp, such as sd *B. licheniformis* [94,95].

In 2020, Almeida and coworkers [90] synthesized Adepamycin, a peptide based on a trypsin inhibitor from *A. pavonina* seeds, showing antimicrobial activity by damaging the membrane integrity of various pathogens without displaying toxicity against human red blood cells. Additionally, Costa et al. [96] explored the action of a peptide from *J. curcas* seeds, JcTI-I, on proteases from *S. enterica* and *S. aureus*, suggesting an alternative antimicrobial mechanism of trypsin inhibitors. Further studies, such as those by Mehmood et al. [62], Rodrigues et al. [91], and Wang et al. [97], have further uncovered the bacteriostatic properties of trypsin inhibitors and their efficacy against bacterial pathogens.

Notably, two recent studies have unveiled novel PPIs with significant antimicrobial potential from Moringa and Salvia plants. In 2021, Cotabarren et al. [98] reported the purification and characterization of the first phytocystatin isolated from *M. oleifera* seeds (MoPI). MoPI, with a molecular mass of 19 kDa, shows higher physicochemical stability against acidic pHs and high temperatures. The study highlighted MoPI as one of the most potent cysteine PPI identified to date, exhibiting Ki and IC50 values within the nanomolar range. Additionally, MoPI has been recognized as a multifunctional molecule, showing robust antimicrobial activity against human pathogens, including *E. faecalis* and *S. aureus*, as well as significant anticoagulant activity. More recently, a trypsin inhibitor named ShTI was isolated and characterized from mucilage and fat-free chia seeds (*S. hispanica* L.). This inhibitor demonstrated exceptional thermostability and wide pH tolerance, likely due to its disulfide bridge structure [99]. ShTI exhibited antibacterial activity against *S. aureus*, including strains resistant to methicillin. However, it showed no significant effects against the Gram-negative bacteria tested. As such, ShTI presents itself as a promising candidate for standalone use or in combination therapy with oxacillin for managing *S. aureus* infections.

### 5.2. Plant Protease Inhibitors with Antifungal Activity

Phytopathogenic fungi produce extracellular proteinases that play an active role in the development of plant diseases [101]. In response to the attack, plants synthesize inhibitors to inactivate these proteinases [21]. This phenomenon was observed for the first time in tomatoes infected by *Phytophthora infestans* [102], in which it was found that high levels of trypsin and chymotrypsin inhibitors correlated with the resistance of the plants to the pathogen [103]. A similar strategy is also used by *S. tuberosum* spp., which produce various PPIs with strong antifungal activity [100]. Importantly, these bioactive molecules extend their fungicidal efficacy beyond phytopathogenic strains to include numerous human pathogens, highlighting their broad-spectrum antimicrobial potential.

Among the inhibitors mentioned in the previous section, Potide-G and PG-2, both Kunitz-type serine PPIs, have been shown to effectively inhibit the growth of several pathogenic fungal strains, such as *C. albicans* [26,92]. Similarly, AFP-J, isolated from *S. tuberosum* cv. L. Jopung [63], targets chymotrypsin, pepsin, and trypsin, exhibiting significant antifungal activity against *C. albicans*, *T. beigelii*, and *S. cerevisiae*. Additional Kunitz-type trypsin and chymotrypsin inhibitors with antifungal properties have been isolated and characterized. Among these, ApTIA, ApTIB, and ApTIC were extracted from the seeds of the Brazilian plant *A. plumosa*, demonstrating notable antifungal activity against pathogenic fungi, including *A. niger*, *T. paradoxa*, and *Colletotrichum* spp. Their mechanism of action is believed to involve the inhibition of serine proteases secreted by these fungi [77]. Moreover, Kunitz-type inhibitors such as RfIP-1 and PDInhibitor have been identified from *R. frangula* and *Conyza dioscoridis*, respectively. These inhibitors have exhibited strong antifungal activity against various fungal strains, including the commercial aerobic filamentous fungus *A. oryzae*. Furthermore, the protease inhibitor from *Coccinia grandis* has demonstrated an antifungal effect on *C. albicans*, *Mucor indicus*, *Penicillium notatum*, *Aspergillus flavus*, *Aspergillus oryzae*, and *Cryptococcus neoformans* [83].

From the Fabaceae family, the tropical trees *I. edulis* and *I. laurina* have also been explored as sources of novel bioactive molecules with antifungal properties. Two distinct Kunitz-type trypsin inhibitors, ILTI and IETI, exhibiting anticandidal activity, were isolated, and their activities characterized [76,84]. ILTI demonstrated potent inhibitory enzymatic activity against trypsin and significantly inhibited the growth of *C. tropicalis* and *C. buinensis*, causing changes in the membrane and permeabilization of yeast cells, as well as the production of reactive oxygen species (ROS). However, this inhibitor did not show efficacy against human pathogenic bacteria such as *E. coli*, *S. aureus*, and *K. pneumoniae*. IETI, characterized by a single trypsin-reactive site stabilized by a disulfide bridge and notable pH and thermal stability, displayed growth-inhibitory activity towards *Candida* spp., including *C. buinensis* and *C. tropicalis*. The yeast inhibitory action of these inhibitors involves multiple mechanisms, including protease inhibition, plasma membrane disruption affecting cell viability, oxidative stress targeting mitochondria, and inducing apoptosis in yeast by blocking critical serine peptidases (e.g., metacaspases) and a nuclear apoptosis mediator [104]. It was also observed that API from *Albizia amara* had an inhibitory effect on *C. albicans* [87], and Adepamycin from *Adenanthera pavonina* inhibited the growth of *C. albicans* and *C. tropicalis* [87,88]. Only recently, Cotabarren et al. [93] reported the purification and characterization of YBPTI, a thermostable trypsin inhibitor from yellow pepper (*C. annuum* L.) seeds with dual antifungal and hypoglycemic properties. The purified inhibitor showed potent and specific in vitro activity against *C. albicans* and showed no cytotoxicity against human Hela cells (ATCC CCL-2). Furthermore, the inhibitor also exhibited α-1,4-glucosidase inhibition activity, positioning this inhibitor as one of the first plant-derived molecules with such a particular dual combination of biological activities, demonstrating that this new inhibitor could be potentially used as an antifungal agent in pharmaceutical preparations to prevent invasive *Candida* infections.

In summary, plant-derived PPIs represent a valuable resource for the development of novel antimicrobial agents. The inherent stability, broad-spectrum antimicrobial efficacy, multifaceted functionality, and minimal toxicity to human cells position these natural compounds as promising candidates for advancing the treatment of microbial infections. Additionally, some of these inhibitors combine both strong antifungal activity and antibacterial properties, potentially serving as dual-purpose antimicrobial molecules.

## 6. Concluding Remarks and Future Perspectives

The worldwide rise of AMR and its impacts on plant, animal, and human health represent a formidable challenge to global health, with the rate of resistance development outpacing the introduction of new antibiotics. The widespread and sometimes indiscriminate use of antimicrobials in medicine, agriculture, and industry has accelerated this process, driving the emergence and spread of AMR as one of the world’s most pressing public health issues. Currently, infections by AMR pathogens are causing over 700,000 deaths per year. It is estimated that by 2050, AMR infections will be responsible for 10 million deaths annually worldwide, significantly impacting the global economy [13,105,106]. Due to its great complexity, it must be approached from different disciplines to frame it within the One Health approach. Thus, several countries have implemented national action plans to combat antibiotic-resistant microbes, following the guidelines of the FAO [107] and the WHO [14]. In this context, natural products, particularly from plant sources, offer a vast repository of chemical diversity that could lead to the discovery of novel antimicrobial agents [2].

Over the last 20 years, a number of natural PPIs with antibacterial and antifungal properties have been isolated from diverse plant sources. In this review, we aim to provide a comprehensive analysis of these molecules and insight into their specific activities, with a special focus on exploring PPIs as relevant agents against biomedical pathogens. The review has described the classification and the diverse mechanisms of action of these proteinaceous molecules, which exhibited a wide range of activities, ranging from disrupting microbial cell membranes to inhibiting vital enzymatic pathways. Taken together, these mechanisms provide novel opportunities for combating pathogens that have evolved resistance to current treatments.

However, to date, only a limited number of antimicrobial peptides (AMPs), such as nisin, gramicidin, polymyxins, and daptomycin have been adopted for clinical use [27]. There remains a significant need for extensive research to identify and enhance potential plant-derived PPIs with therapeutic significance. In this context, efforts have been made to develop novel computational tools aimed at predicting antimicrobial peptide sequences [108]. Moreover, such in silico approaches can also be utilized to improve molecules already known for their antimicrobial activity. Structure-based drug discovery strategies, for instance, might employ the known three-dimensional structures of target proteins to design molecules with enhanced biological activities [109]. This rational design approach will facilitate the development of novel, engineered molecules with superior antimicrobial properties.

In summary, this review illustrates the enormous potential of the PPIs from plant species in medicine. Their broad-spectrum antimicrobial activities, combined with their specificity and minimal toxicity towards non-target organisms, highlight their value as a sustainable and effective alternative to conventional synthetic chemicals. This, coupled with their multifunctionality, positions these molecules as integral components of the next generation of antimicrobial agents.

## Figures and Tables

**Table 1:** Peptide protease inhibitors derived from plants with antimicrobial activity.

Plant Family	Plant Source	Name	Type of PPI	MW (kDa)	Activity	Target Microorganisms	Ref.
*Fabaceae* (*Leguminosae*)	*Inga edulis*	Inga Edulis Trypsin Inhibitor (IETI)	Kunitz	19.7	Antifungal	*Candida tropicalis* and *Candida buinensis*	[76]
	*Inga laurina*	Inga Laurina Trypsin Inhibitor (ILTI)	Kunitz	20	Antifungal	*C. tropicalis* and *C. buinensis*	[84]
	*Acacia plumosa*	Acacia plumosa Trypsin Inhibitor (ApTI A, B, C)	Kunitz	20	Antifungal	*Aspergillus niger*, *Thielaviopsis paradoxa*, and *Colletotrichum* spp.	[77]
	*Acacia nilotica*	Acacia nilotica Trypsin Inhibitor (AnTI)	Trypsin inhibitor	21	Antibacterial	*G+*: *Staphylococcus aureus and Bacillus subtilis*, *G−: Escherichia coli*, and *Pseudomonas aeruginosa*	[62]
	*Albizia amara*	Proteinaceous protease inhibitor (API)	Unknown	49	Antibacterial	*G+: B. subtilis* *G−: P. aeruginosa*	[87]
					Antifungal	*C. albicans*	
	*Luetzelburgia auriculata*	Luetzelburgia auriculata Bowman-Birk protease inhibitor (LzaBBI)	Bowman-Birk	14.3	Antibacterial	*G+: S. aureus*	[88]
	*Cassia fistula*	Fistulin	Trypsin inhibitor	4	Antibacterial	*G+: S. aureus*, and *B. subtilis* *G−: Klebsiella pneumoniae, E. coli*, and *P. aeruginosa*	[89]
	*Adenanthera pavonina*	Adepamycin	Trypsin inhibitor	2.4	Antibacterial	*G+: S. aureus* *G−: E. coli*, *Klebsiella oxytoca*, *K. pneumoniae*, and *P. aeruginosa*	[90,91]
					Antifungal	*C. albicans* and *C. tropicalis*	
*Solanaceae*	*Solanum tuberosum* L. Cv. Golden Valley	Potide-G	Kunitz	5.57	Antibacterial	*G+: S. aureus*, and *Listeria monocytogenes* *G−: E. coli*	[26]
					Antifungal	*C. albicans* and *Rhizoctonia solani*	
	*S. tuberosum* L. Cv. Gogu Valley	Peptide G2 (PG-2)	Kunitz	3.2	Antibacterial	*G+: S. aureus*	[92]
					Antifungal	*C. albicans*	
	*S. tuberosum* L. Cv. L. Jopung	Antifungal protein J (AFP-J)	Kunitz	13.5	Antifungal	*C. albicans*, *Trichosporon beigelii*, and *Saccharomyces cerevisiae*	[63]
	*Capsicum annuum* L.	Yellow Bell Pepper Trypsin Inhibitor (YBPTI)	Trypsin inhibitor		Antifungal	*C. albicans*	[93]
*Cucurbitaceae*	*Coccinia grandis* (L.) *Voigt.*	*Coccinia grandis* (L.) inhibitor	Protease inhibitor	14.3	Antibacterial	*G+: S. aureus*, and *B. subtilis* *G−: K. pneumoniae*, *Proteus vulgaris*, and *E. coli*	[83]
					Antifungal	*C. albicans*, *Mucor indicus*, *Penicillium notatum*, *Aspergillus flavus Aspergillus oryzae*, and *Cryptococcus neoformans*	
*Rhamnaceae*	*Rhamnus frangula*	RflP-1	Kunitz	22.5	Antibacterial	*G*+: *Bacillus* sp. and *Bacillus licheniformis*	[94,95]
*Euphorbiaceae*	*Jatropha curcas*	Jatropha curcas Trypsin inhibitor I (JcTI-I)	Trypsin inhibitor	10.2	Antibacterial	*G+: S. aureus* *G−: Salmonella enterica*	[96]
*Asteraceae*	*Helianthus annuus*	Sunflower Trypsin Inhibitor 1 (SFTI1)	Trypsin inhibitor		Antibacterial	*G+: Staphylococcus epidermidis*, *S. aureus*, and *Enterococcus faecalis* *G−: E. coli*, *P. aeruginosa*, and *Salmonella typhimurium*	[97]
*Moringaceae*	*Moringa oleifera*	Moringa oleifera Protein Inhibitor (MoPI)	Phytocystatin	19	Antibacterial	*G+: S. aureus*, and *E. faecalis*	[98]
*Lamiaceae*	*Salvia hispanica*	Salvia hispanica Trypsin Inhibitor (ShTI)	Trypsin inhibitor	11	Antibacterial	*G+: Methicillin-resistant* *S. aureus*	[99]

## Data Availability

Not applicable.
